# Sodium-Glucose Cotransporter 2 Inhibitor and a Low Carbohydrate Diet Affect Gluconeogenesis and Glycogen Content Differently in the Kidney and the Liver of Non-Diabetic Mice

**DOI:** 10.1371/journal.pone.0157672

**Published:** 2016-06-21

**Authors:** Kuralay Atageldiyeva, Yukihiro Fujita, Tsuyoshi Yanagimachi, Katsutoshi Mizumoto, Yasutaka Takeda, Jun Honjo, Yumi Takiyama, Atsuko Abiko, Yuichi Makino, Masakazu Haneda

**Affiliations:** Division of Metabolism an Biosystemic Science, Department of Internal Medicine, Asahikawa Medical University, Asahikawa, Hokkaido, Japan; East Tennessee State University, UNITED STATES

## Abstract

A low carbohydrate diet (LCHD) as well as sodium glucose cotransporter 2 inhibitors (SGLT2i) may reduce glucose utilization and improve metabolic disorders. However, it is not clear how different or similar the effects of LCHD and SGLT2i are on metabolic parameters such as insulin sensitivity, fat accumulation, and especially gluconeogenesis in the kidney and the liver. We conducted an 8-week study using non-diabetic mice, which were fed ad-libitum with LCHD or a normal carbohydrate diet (NCHD) and treated with/without the SGLT-2 inhibitor, ipragliflozin. We compared metabolic parameters, gene expression for transcripts related to glucose and fat metabolism, and glycogen content in the kidney and the liver among the groups. SGLT2i but not LCHD improved glucose excursion after an oral glucose load compared to NCHD, although all groups presented comparable non-fasted glycemia. Both the LCHD and SGLT2i treatments increased calorie-intake, whereas only the LCHD increased body weight compared to the NCHD, epididimal fat mass and developed insulin resistance. Gene expression of certain gluconeogenic enzymes was simultaneously upregulated in the kidney of SGLT2i treated group, as well as in the liver of the LCHD treated group. The SGLT2i treated groups showed markedly lower glycogen content in the liver, but induced glycogen accumulation in the kidney. We conclude that LCHD induces deleterious metabolic changes in the non-diabetic mice. Our results suggest that SGLT2i induced gluconeogenesis mainly in the kidney, whereas for LCHD it was predominantly in the liver.

## Introduction

Carbohydrates are essential nutrients, which maintain homeostasis in the body and are a major energy source. Once taken orally, carbohydrates are digested into monosaccharides such as glucose, absorbed from the gut, delivered to the liver and then circulated to the peripheral organs including the kidneys. In normal conditions, glucose undergoes filtration but is entirely re-absorbed from the kidneys. Although carbohydrates are obviously requisite, the chronic excess intake of carbohydrates can induce obesity and subsequently type 2 diabetes mellitus (T2DM) [1–5]. Obesity is highly associated with risks of hypertension, hyperlipidemia, and cardiovascular disease [6–8]. In addition, T2DM is strongly coupled with microvascular complications such as retinopathy and diabetic kidney disease.

It is widely appreciated that diet is a key approach in maintaining adequate body weight (BW) especially for obese people with complications. Total calorie restriction is one common approach to dieting, with another being a nutrition-oriented restriction such as a low-carbohydrate diet (LCHD) or a low-fat diet. A LCHD leads to less glucose influx to the portal vein from the gut, suppresses postprandial elevation of glucose, possibly resulting in amelioration of obesity and insulin resistance [9–11].

A novel class of anti-diabetic drugs, sodium glucose cotransporter type 2 inhibitors (SGLT2i) not only show convincing glucose-lowering effects but also exhibit promising effects on metabolic disorders such as obesity and insulin resistance [12–15]. SGLT2is exert their therapeutic activity independent of insulin action, by facilitating glucose excretion through the kidney. Clinical data shows that SGLT2is have the potential to improve glycemia without the risk of hypoglycemia and promote weight loss [16–18].

LCHD and SGLT2i might similarly decrease glucose utilization, increase fat oxidation and ketone production and shift the whole body towards catabolism. However, glucose utilization is restricted in two different ways: a LCHD limits glucose influx from the gut, while SGLT2i enhances urinary glucose disposal. It is presumed that LCHD lowers portal glucose levels and diminishes hepatic glucose uptake. In contrast, SGLT2is likely do not manipulate portal glucose levels, but may manipulate metabolic changes in the kidney, such as gluconeogenesis or lipid oxidation.

In the present study, we investigated the effects of LCHD, SGLT2i, and their combination using non-obese and non-diabetic mice. We compared glucose tolerance, insulin secretion, insulin sensitivity, food consumption, and adipose accumulation. Then, we investigated how these treatments regulate gluconeogenesis, glycolysis, fatty acid synthesis and β-oxidation, and how they contributed to glycogen and fat storage in the liver and the kidney.

## Materials and Methods

### Animals and experimental design

Six-week-old male C57Bl-6J mice were purchased from Charles River Laboratories Japan Inc. (Yokohama, Japan). This study was carried out in strict accordance with the guide for the care and use of laboratory animals at Asahikawa Medical University. The protocol was approved by Asahikawa Medical University Animal Research Committee (No15070, No14063, and No16129).

After two days of acclimatization, mice were fed a normal carbohydrate diet (NCHD) or LCHD ad libitum for 8 weeks with or without SGLT2i treatment. Mice were randomized into four groups (each n = 6–10): the LC group was fed with LCHD, the NC+Ipra group was fed with NCHD and treated with a SGLT2i ipragliflozin, the LC+Ipra combined group was fed with LCHD and treated with Ipragliflozin, and the NC group was fed with NCHD, respectively. Table 1 shows the formulas of the experimental diets used in this study (Cat#D10001, Cat#D14012301, Research Diet, New Brunswick, NJ, USA). Briefly, NCHD consists of carbohydrate:protein:fat (C:P:F) = 68:21:12% kJ and LCHD consists of C:P:F = 16:40:44% kJ. Ipragliflozin was kindly provided by Astellas Pharma Inc. (Ibaraki, Japan) and was suspended in a 0.5% methylcellulose solution at the time of use. Ipragliflozin (3 mg/kg body wt) or NaCl (154 mmol/l) solution was administered daily by oral gavage between 4 and 6 pm. Body weights were determined and non-fasted blood glucose levels were measured with a Onetouch Ultra glucometer (Lifescan, Tokyo, Japan) at the start of the study and twice a week thereafter until day 56. Food intake was measured every day during the experiment. For fasting studies, mice were fasted overnight (18 h) before analysis.

**Table 1:** Diets composition.

Product #	D10001 (NCHD)		D14012301 (LCHD)	
	g%	kJ%	g%	kJ%
Protein	20	87,8	47	167,4
Carbohydrate	66	284,5	19	66,9
Fat	5	50,2	23	184,1
Total		100		100
kJ/g	16,3		19,7	
**Ingredient**	**g**	**kJ**	**g**	**kJ**
Casein	200	3347,2	384	6426,6
DL-Methionine	3	50,2	6	100,4
Corn Starch	150	2510,4	50	836,8
Maltodextrine	0	0	100	1673,6
Sucrose	500	8368	0	0
Cellulose, BW200	50	0	50	0
Corn oil	50	1882,8	50	1882,8
Lard	0	0	139	5234,2
Mineral Mix S10001	35	0	35	0
Vitamin Mix V10001	10	167,4	10	167,4
Choline bitartrate	2	0	2	0
FD&C red dye #40	0	0	0,05	0
**Total**	**1000**	**16326**	**826,05**	**16321,8**

### Oral glucose tolerance test (OGTT) and intraperitoneal insulin tolerance test (ITT)

OGTTs were performed at day 53 of the study. Glucose (25 wt/vol%) was orally administered (2 mg/g body wt) after 18 h of fasting. Blood was obtained from the tail vein and blood glucose levels were measured at 0, 15, 30, 60, 90 and 120 min after gavage. Blood samples were collected into heparinized tubes at 0 and 15 min and centrifuged for the analysis of glucose-stimulated blood insulin. ITTs were performed at day 46. Mice were fasted for 4 h and then injected with insulin (Novolin R (Novo Nordisk), 0.6 U/kg body wt) intraperitoneally. Blood glucose levels were measured at 0, 30, 60 and 90 min after injection. Values are shown as % blood glucose compared to blood glucose levels at 0 min.

### Biochemical analysis

Non esterified fatty acids (NEFA) and triglyceride (TG) levels were measured at day 39 by enzymatic colorimetric assays (LabAssay NEFA Kit and LabAssay Triglyceride Kit; Wako Pure Chemical Industries, Osaka, Japan) after overnight fasting. Plasma insulin and glucagon levels were measured using commercial ELISA kits (Mouse Insulin ELISA Kit; Morinaga Institute of Biological Science, Yokohama, Japan and Glucagon ELISA Kit; Mercodia AB, Uppsala, Sweden), following the manufacturer’s instructions. Ketone bodies (3-hydroxybutyrate) were also measured at day 39 by an enzymatic colorimetric assay (Ketorex Kit; Sanwa Kagaku Kenkyusho, Nagoya, Japan) after overnight fasting. HbA1c were measured using a DCA Vantage Analyzer (Siemens, Munich, Germany) in blood collected from the tail vein on day 53.

### Tissue collection

On the last day of the study, day 56, non-fasted mice were sacrificed under whole body inhalable anesthesia (Isofluorane 2%). The kidney and liver were rapidly removed, and part were flash frozen in liquid nitrogen for RNA extraction and tissue glycogen/TG content, and the remaining part were fixed overnight for histological analysis. Epididymal fat pads were dissected from each animal and weighed to evaluate visceral fat accumulation.

### Quantitative analysis of mRNA expression in the kidney and the liver

Total RNA was isolated from the kidney and the liver tissues using Trizol Reagent (Life Technologies, CA, USA). cDNA was synthesized using a SuperScript First-Strand Synthesis System for RT-PCR (Invitrogen, CA, USA) according to the manufacturer’s instructions. Quantitative RT-PCR was performed using *Rpl37A* as an internal standard on the basis of TaqMan Gene Expression Assays (Applied Biosystems, Foster City, CA). Probes for TaqMan Gene Expression Assays are presented in Table 2.

**Table 2:** Taqman (R) gene expression assays annotation.

Assay ID	Gene symbol	Gene name	NCBI assembly build number
Mm01546394_s1	*Rpl37a*	Ribosomal protein L37a	NM_009084.4
Mm00839363_m1	*G6pc*	Glucose-6-phosphotase	NM_008061.3
Mm01247058_m1	*Pck1*	Phosphoenolpyruvate carboxikinase	NM_011044.2
Mm00614545_m1	*Acad11*	Acyl-Coenzyme A dehydrogenase family, member 11	NM_175324.3
Mm00662319_m1	*Fasn*	Fatty acid synthase	NM_007988.3
Mm01289790_m1	*Pygl*	Glycogen phosphorylase	NM_133198.2
Mm01962575_s1	*Gys1*	Glycogen synthase 1, muscle	NM_030678.3
Mm01267380_m1	*Gys2*	Glycogen synthase 2, liver	NM_145572.2
Mm00490671_01	*Foxo1*	Forkhead box 01	NM_019739.3
Mm00501607_m1	*Creb1*	cAMP responsive element binding protein 1	NM_009952.2

### Tissue TG and glycogen content in the liver and the kidney

Tissue glycogen content was measured by colorimetric assay using the Glycogen Assay Kit II (Abcam, Cambridge, UK). To measure tissue TG content, approximately 100 mg of liver or kidney tissue was digested overnight with ethanolic potassium hydroxide (2 parts ethanol: 1 part 30 wt/vol% KOH) at 55°C, and the digested tissues were extracted twice with 50 vol/vol% ethanol. After neutralization with 1 mol/L magnesium chloride, the supernatant was used for TG measurement using a TG kit (LabAssay TG Kit; Wako Pure Chemical Industries, Osaka, Japan).

### Histological analysis

The collected kidney and the liver tissues fixed in 4% paraformaldehyde in PBS overnight at 4°C and then embedded in paraffin blocks. Embedded tissue was sliced into 3 μm sections and deparaffinised with a series of xylene and ethanol. We performed Best carmine staining [19]. Slides were incubated with carmine solution for 1 h, after which they were washed, mounted and observed under microscopy (BZ-8100; Keyence, Osaka, Japan), and digital images were collected.

### Statistical analysis

All data are expressed as mean ± SEM from repeated experiments. The statistical analyses were performed by one-way ANOVA or Student’s t-test. For the ANOVA procedures, Bonferroni tests were used to establish differences between groups. Statistical significance was set at p < 0.05.

## Results

### Despite higher energy intake in both the LC and NC+Ipra groups, only the LC group developed insulin resistance and obesity

**Fig 1:**
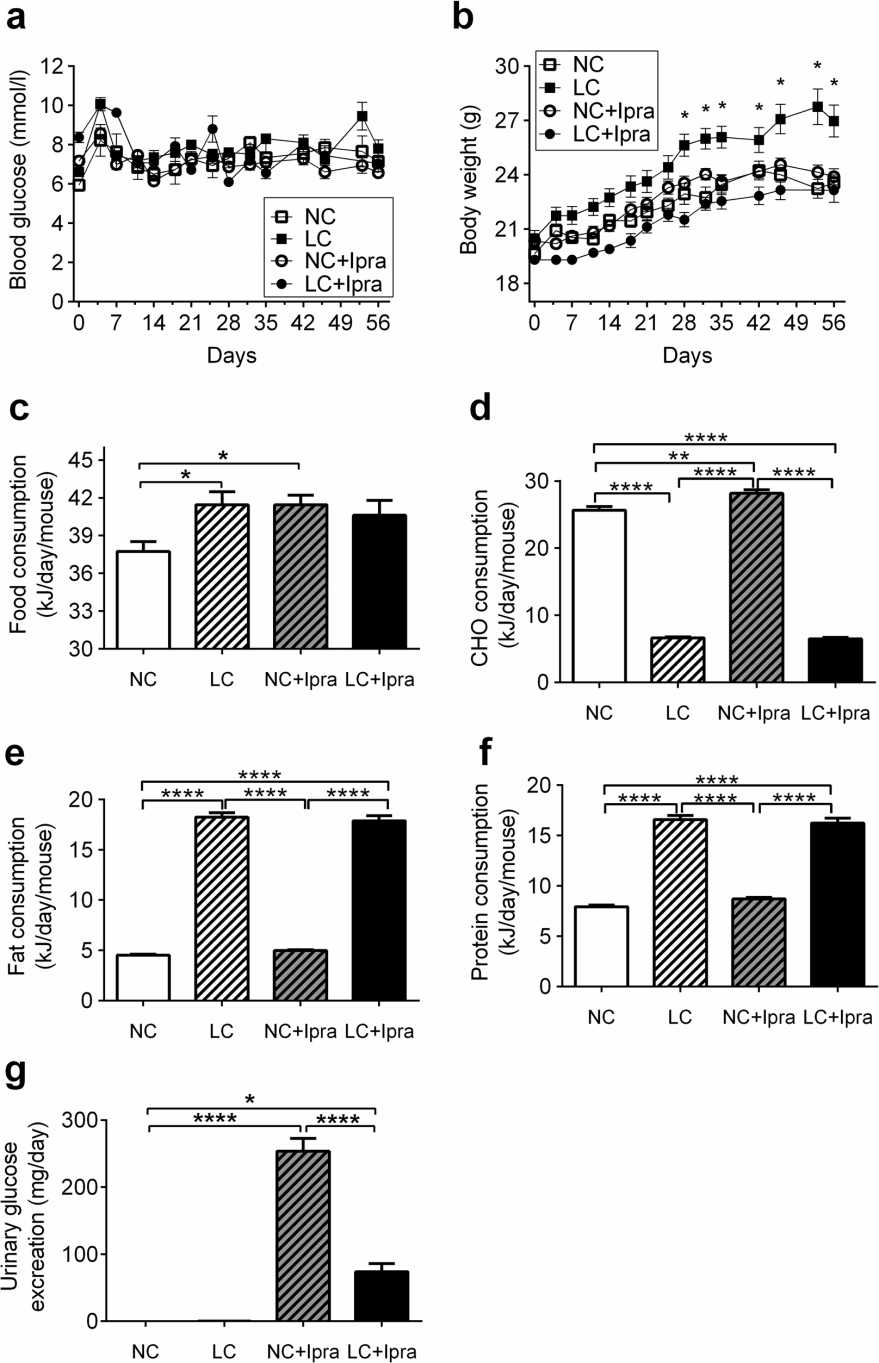
The effect of LCHD and SGLT2i on body weight and glycemia. Body weight (a), non-fasted glycemia (b), average mean daily food intake per mouse (c), average mean daily carbohydrate intake per mouse (d), average mean daily fat intake per mouse (e), average mean daily protein intake per mouse (f), urinary glucose excretion at day 40 (g). n = 6–10. Data are presented as means ± SEM. *p < 0.05, **p<0.01, ****p < 0.0005 vs NC. White squares, NC; black squares, LC; white circles, NC+Ipra; black circles, LC+Ipra; white bars, NC; hatched white bars, LC; hatched grey bars, NC+Ipra; black bars, LC+Ipra.

In all groups, mice gained body weight during the study (Fig 1A). There was no difference in non-fasted glycemia among the groups during the study (Fig 1B). However, the LC group showed significantly higher body weight compared to the other three groups at week 4 and thereafter. Daily food intake in LC and NC+Ipra groups were similar, but significantly higher than in the NC group (LC: 41.5 ± 1.0; NC+Ipra: 41.4 ± 0.8 kJ vs NC: 37.7 ± 0.2 kJ, p < 0.05) (Fig 1C). We calculated average daily carbohydrate (d), fat (e) and protein (f) intakes according to diet composition formulas and the carbohydrate consumption in the NC+Ipra group was significantly higher compared that in the NC group. The fat intake in LCHD treated groups showed more than three times higher than that of NCHD fed groups. Glucosuria was induced by a daily treatment of ipragliflozin both in the NC+Ipra and LC+Ipra groups, and no glucosuria was observed in the NC and LC groups (Fig 1G). Dietary carbohydrate restriction significantly reduced ipragliflozin-induced glucosuria (NC+Ipra: 253.6 ± 19.2 mg/day; LC+Ipra: 73.9 ± 12.2 mg/day, p < 0.0001). Daily energy utilization, which is calculated food consumption minus the urinary energy loss, was similar between the NC, NC+Ipra and LC+Ipra groups (NC: 37.7 ± 0.2 kJ; NC+Ipra: 37.3 ± 0.8 kJ; LC+Ipra: 39.4 ± 1.0).

Metabolic variables were analyzed at days 39 and 53 (Table 3). The fasting glucose and insulin levels were significantly higher in the LC group compared to the NC and NC+Ipra groups. Fasting glucagon levels were significantly lower in the LC, NC+Ipra and LC+Ipra groups compared to the NC. TG levels were significantly higher in the NC+Ipra group compared to the other groups. There were no differences in NEFA, HbA_1C_ and 3-hydroxybutyrate levels among the groups.

**Table 3:** Plasma variables in the control and LCHD or/and Ipragliflozin treated mice.

Variable	NC	LC	NC+Ipra	LC+Ipra
	n = 6	n = 6	n = 6	n = 6
**Fasted glucose, mmol/L**	2.84±0.1	3.49±0.1^**a**^	2.96±0.1	3.48±0.1^**a**^
**Fasted insulin, ng/ml**	0.44±0.04	0.73±0.05^**a**^	0.41±0.03	0.36±0.03
**Fasted glucagon, pmol/L**	2.65±0.65	0.43±0.15^**a**^	0.55±0.14^**b**^	0.9±0.45^**b**^
**TG, mmol/L**	1,28±0,2	1,61±0,1	1,79±0,1^**b**^	1,08±0,1
**NEFA, mmol/L**	1.28±0.15	1.3±0.12	1.5±0.08	1.04±0.14
**HbA1C, %**	3.7±0.2	3.8±0.03	3.8±0.06	3.4±0.1
**3-hydroxybutyrate, mmol/L**	4.85±1.0	2.34±0.8	5.39±0.3	1.14±0.1

Data represent mean ±SEM

Blood samples were taken at day 39 for TG and NEFA measurements and at day 53 for the other indicated parameters

^a^Statistically significant difference for comparison with the NC group (p < 0.01)

^b^Statistically significant difference for comparison with the NC group (p < 0.05)

### LCHD produced detrimental effects regarding insulin sensitivity

**Fig 2:**
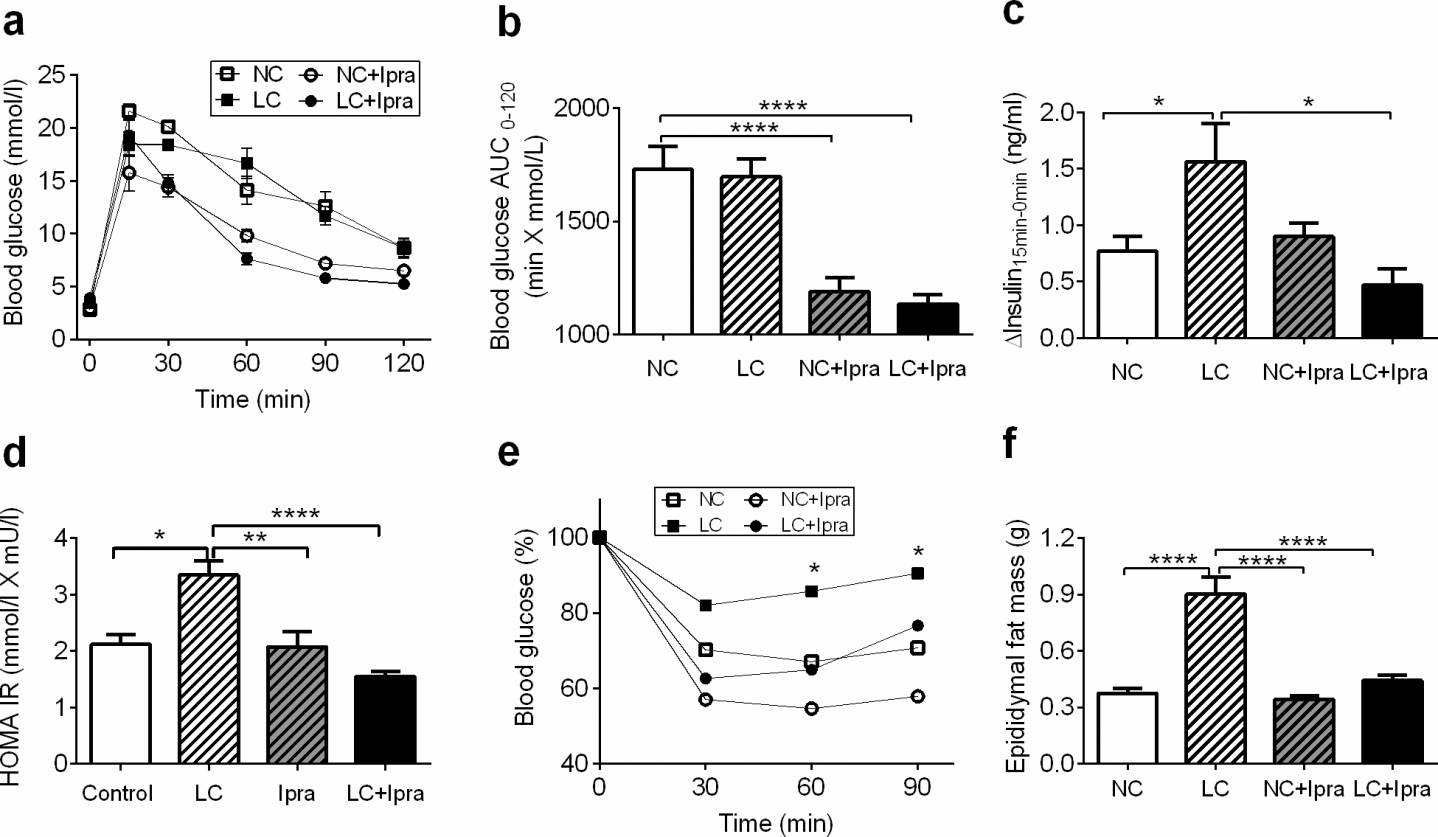
LCHD induced impaired glucose tolerance, insulin resistance and fat accumulation. Blood glucose levels were measured after an oral glucose load (a). Blood glucose AUC in OGTT (b). Glucose stimulated insulin secretion (c) measured during OGTT (Δ-insulin = Insulin_15min_−Insulin _0min_). HOMA IR (d) were calculated according to the formula: HOMA IR = Glucose (mmol/L) X Insulin (mU/L)/22.5. Blood glucose levels decrease after i.p. injection of insulin presented in % (e). Epididymal fat masses were measured at day 56 (f). n = 6–10. Data are presented as means ± SEM. *p < 0.05, **p < 0.01, ***p < 0.005, ****p < 0.0005 vs NC. White squares, NC; black squares, LC; white circles, NC+Ipra; black circles, LC+Ipra; white bars, NC; hatched white bars, LC; hatched grey bars, NC+Ipra; black bars LC+Ipra.

During OGTTs, SGLT2i, but not LCHD reduced glucose excursion after oral glucose administration, compared to the NC group (Fig 2A and 2B). In contrast, the LC group showed a significant increase in glucose-stimulated insulin secretion (Fig 2C), although glucose excursion was comparable to the NC group. HOMA IR was significantly higher in the LC group compared to the NC group (LC: 3.4 ± 0.3; NC: 2.1 ± 0.2 mmol/L X mU/L, p < 0.05) (Fig 2D). Next, we performed ITTs to further evaluate insulin sensitivity. The LC group showed impairment of an insulin-induced glucose lowering, suggesting a LCHD could induce insulin resistance (Fig 2E). Visceral fat as assessed by epididymal fat pad mass, increased significantly only in the LC group (Fig 2F). Calorie-adjusted pair-feeding ameliorated LCHD-induced BW gain, fat deposition and insulin resistance (data not shown).

### SGLT2i activates gluconeogenesis gene expression in the kidney, while LCHD activates expression in the liver

**Fig 3:**
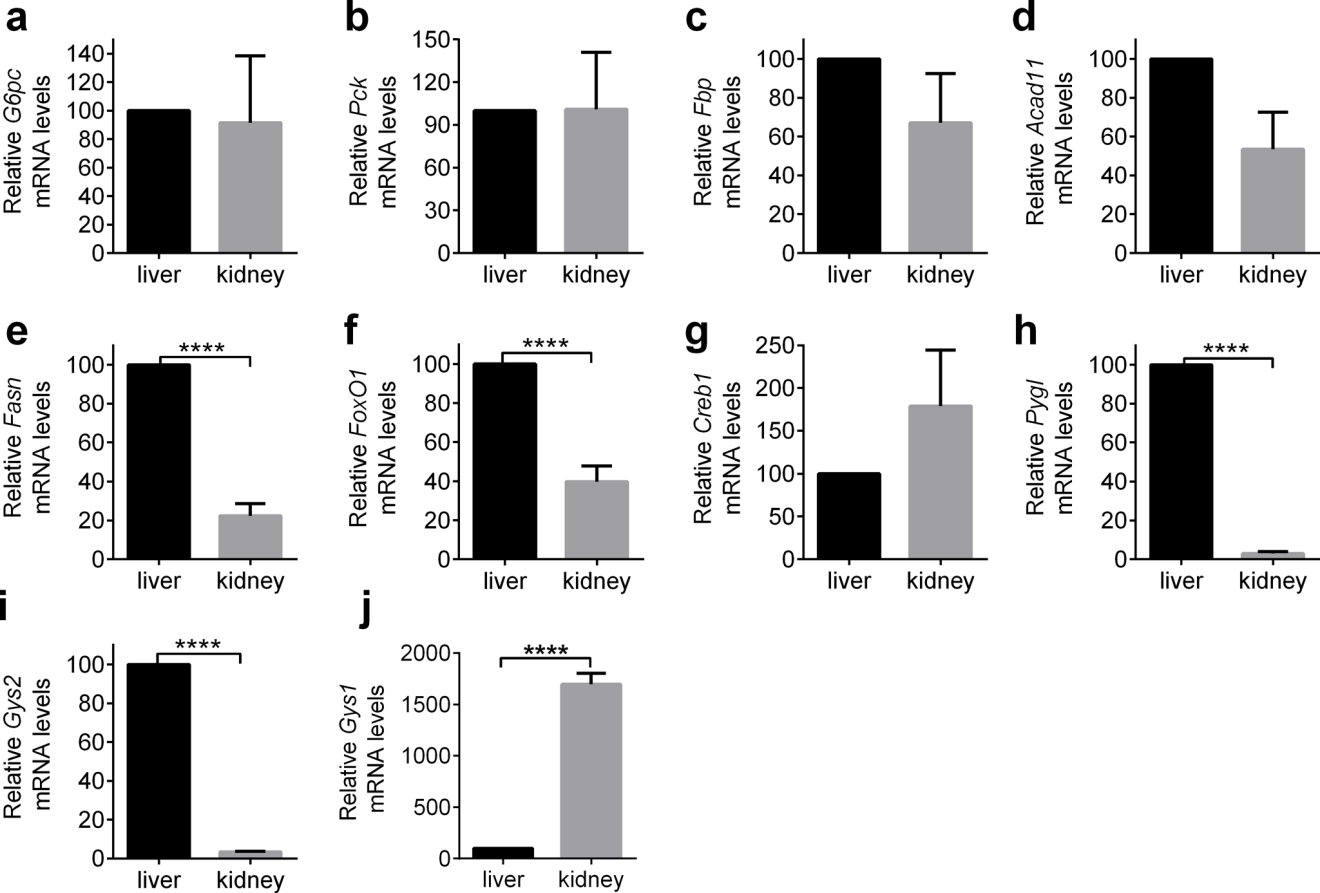
Relative mRNA expression in the liver and the kidney of control mice. mRNA expression in the liver was taken as 100

To investigate further mechanisms of metabolic alterations, we next analyzed the gene expressions of key enzymes related to gluconeogenesis, β-oxidation, fatty acid synthesis, glycogenolysis and glycogen synthesis. We first assessed gene expression of these transcripts in the kidney and the liver of the NC group as control mice (Fig 3A–3I).

**Fig 4:**
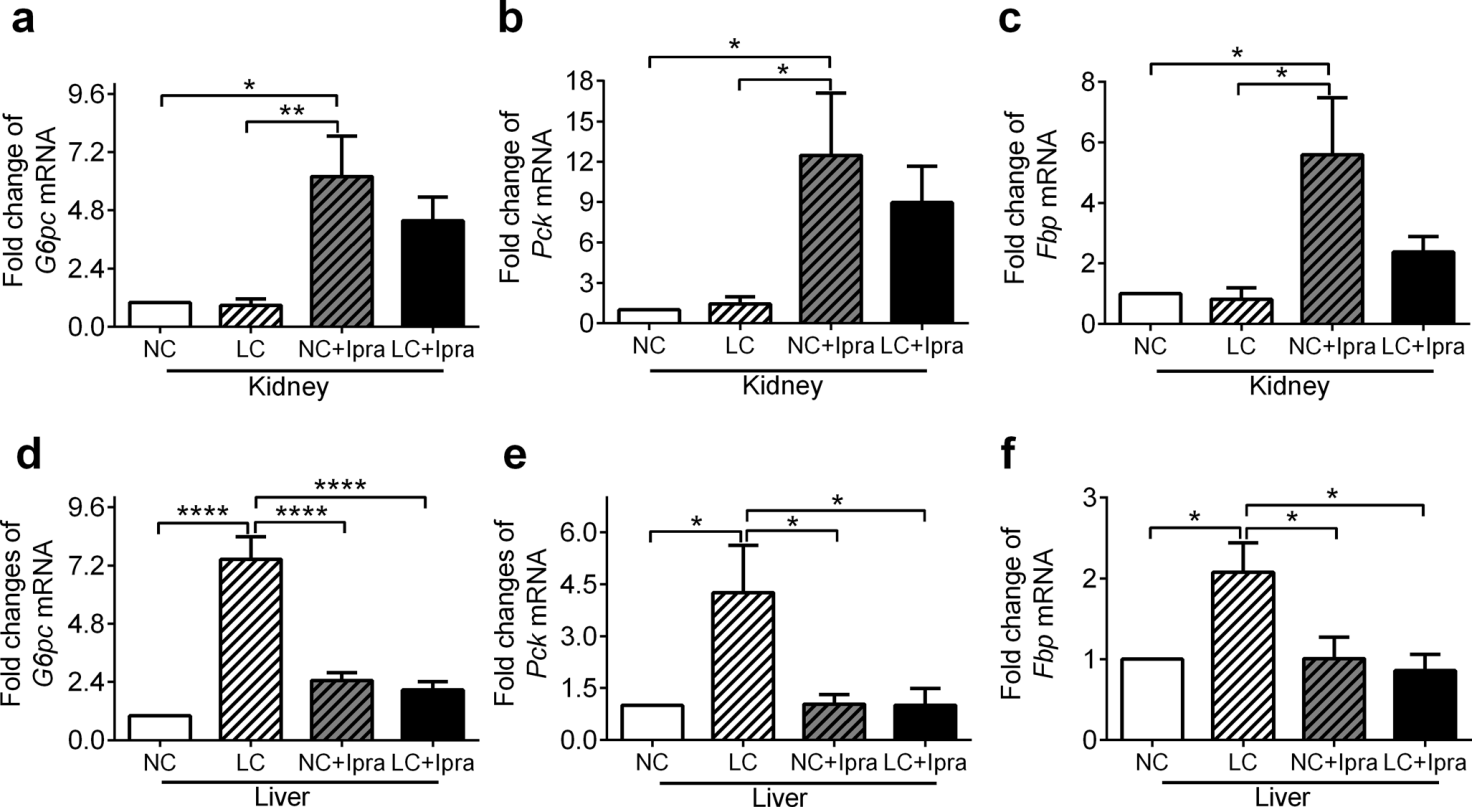
SGLT2i activates gluconeogenesis gene expression in the kidney, while LCHD activates expression in the liver. mRNA expression of mouse *G6pc*, *Pck*, *Fbp* in the kidney (a, b, c) and the liver (d, e, f,) were determined by quantitative RT-PCR. The means ± SEM of mRNA levels related to the NC group are presented. n = 6–10. *p < 0.05, **p < 0.01, ****p < 0.0005 vs NC. White bars, NC; hatched white bars, LC; hatched grey bars, Ipra; black bars LC+Ipra.

Next, we analyzed glucose-6-phosphotase (*G6pc*), phosphoenolpyruvate carboxyl kinase (*Pck1*) and fructose-1,6-bisphosphatase (*Fbp*) gene expressions in the kidney and the liver and compared them among the groups. Ipragliflozin alone simultaneously increased the expression of these transcripts in the kidney (Fig 4A, 4B and 4C), suggesting that SGLT2i could activate renal gluconeogenesis. In contrast, a LCHD may enhance gluconeogenesis in the liver by activation of these genes (Fig 4D, 4E and 4F).

### SGLT2i upregulated both Foxo1and Creb1 genes in the kidney, while LCHD upregulated in the liver

**Fig 5:**
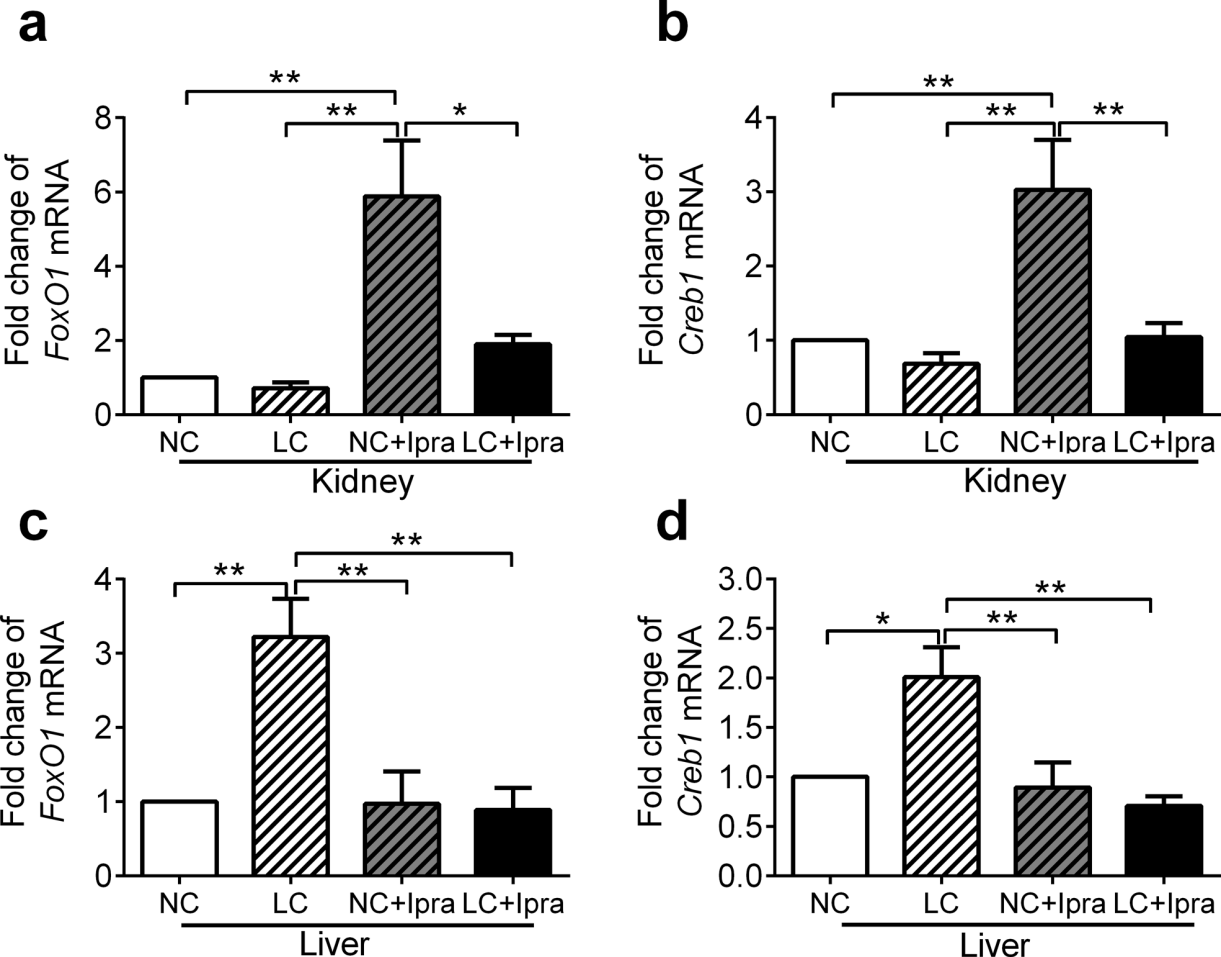
Ipragliflozin upregulated both *Foxo1* and *Creb1* genes in the kidney, while LCHD upregulated in the liver. *Foxo1* and *Creb1* mRNA expression in the kidney (a, b) and liver (c, d) were determined by quantitative RT-PCR. The means ± SEM of mRNA related to NC group are presented. n = 6–10. *p < 0.05, **p < 0.01 vs NC. White bars, NC; hatched white bars, LC; hatched grey bars, NC+Ipra; black bars LC+Ipra.

We investigated gene expressions of forkhead box protein O1 (*Foxo1*) and cAMP responsive element binding protein 1 (*Creb1*), since they are key transcription factors that promote gluconeogenesis via an increase of *G6pc*, *Pck* and *Fbp* expressions. *Foxo1* was upregulated in the kidney of the NC+Ipra group by 5.9 ± 1.5 fold compared to the NC group (p < 0.01) (Fig 5A), whereas in the liver it was upregulated by 3.2 ± 0.5 fold in the LC group compared to the NC group (p < 0.01) (Fig 5C). In the kidney, *Creb1* expression was activated by 3.0 ± 0.7 fold in the NC+Ipra group compared to the NC group (p < 0.01) (Fig 5B), whereas in the liver it was activated in the LC group by 2.0 ± 0.3 fold compared to the NC group (p < 0.05) (Fig 5D).

### SGLT2i treatment reduced liver, but increased kidney glycogen content

**Fig 6:**
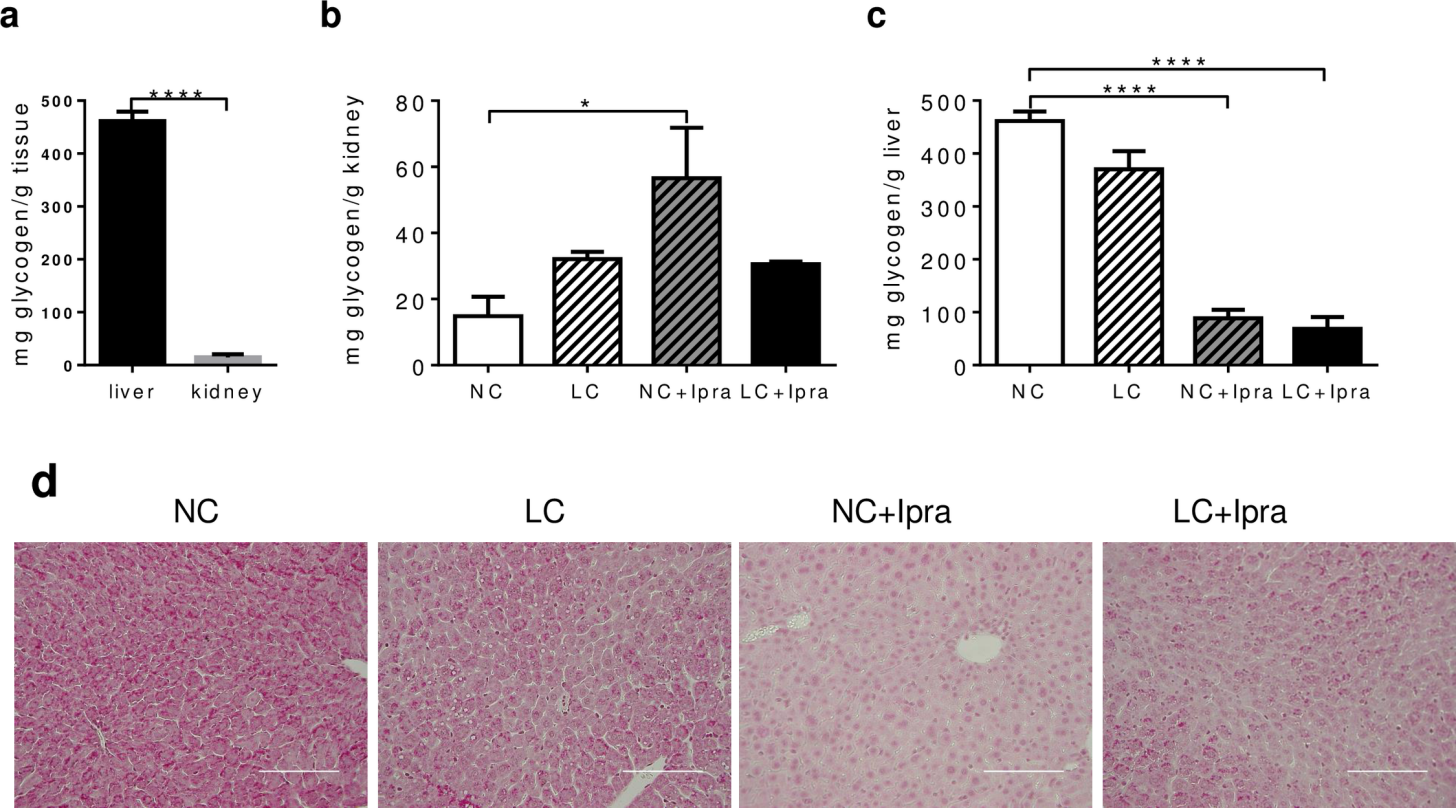
Ipragliflozin treatment reduced liver, but increased kidney glycogen content. Glycogen content in the liver and kidney (a) measured in the NC group. Glycogen content in the kidney (b) and liver (c) in non-fasted mice at day 56. (d) Best carmine staining indicating glycogen storage in the liver. n = 6–10. Data are presented as means ± SEM. Scale bars = 100 μm. *p < 0.05, ****p < 0.0005 vs NC. White bars, NC; hatched white bars, LC; hatched grey bars, Ipra; black bars LC+Ipra.

Next, we compared kidney and liver glycogen content among the groups. In the kidney, SGLT2i alone induced glycogen accumulation (NC: 14.8 ± 5.9; NC+Ipra: 56.6 ± 15.3 mg glycogen/g kidney, p < 0.05) (Fig 6B). However, liver showed markedly lower glycogen content in groups treated with SGLT2i (NC+Ipra: 88.7 ± 16.0 and LC+Ipra: 68.6 ± 22.5, vs NC: 461.6 ± 18.0 mg glycogen/g liver, p < 0.0001). Expectedly, ipragliflozin treatment markedly reduced glycogen staining in the liver (Fig 6D). In contrast, the LC group had a negligible reduction of liver glycogen content (LC: 370.4 ± 34 n.s. vs NC) (Fig 6C).

**Fig 7:**
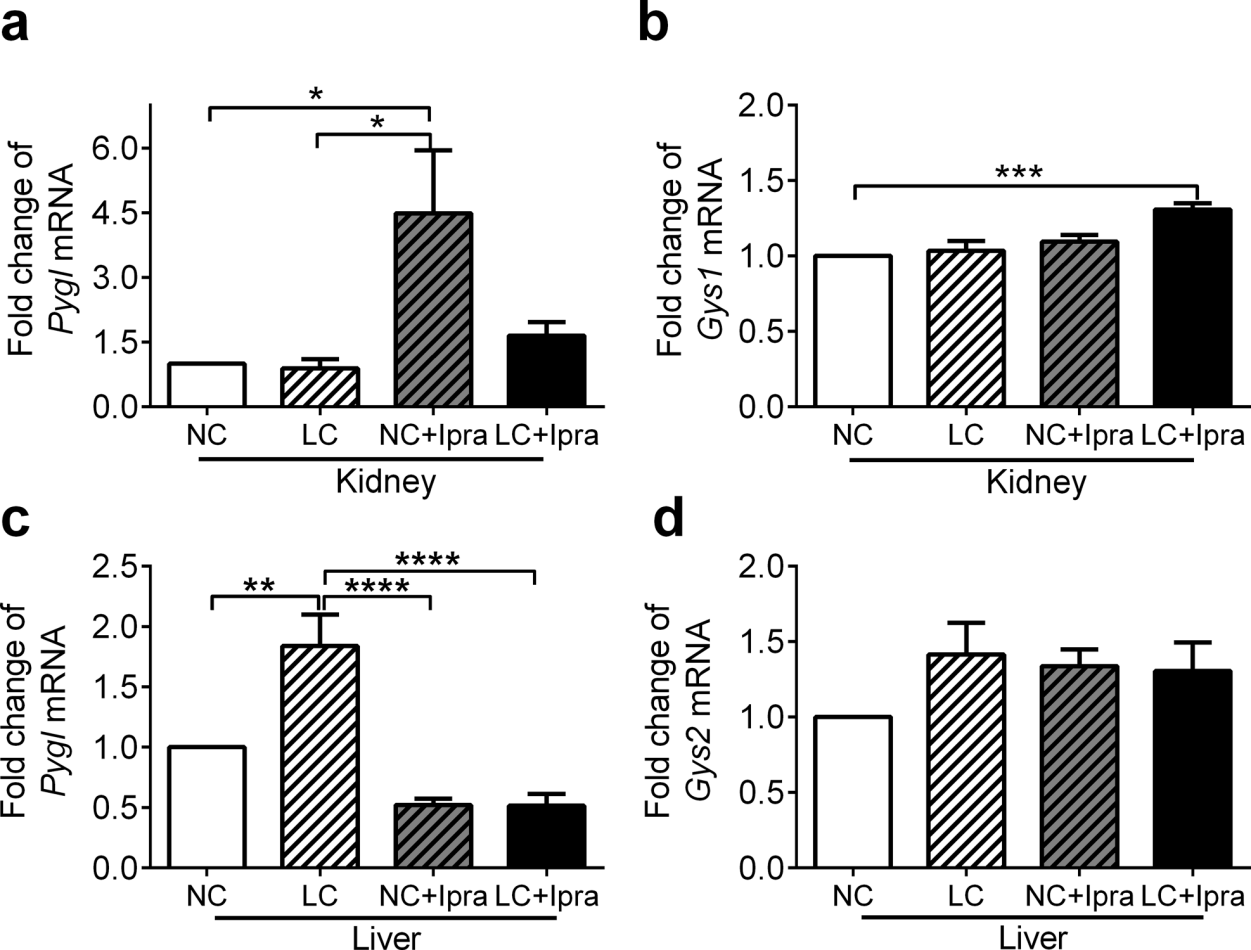
Ipragliflozin increased *Pygl* expression in the kidney, while LCHD increased in the liver. *Pygl* and *Gys1/Gys2* mRNA expression in the kidney (a, b) and in the liver (c, d) were determined by quantitative RT-PCR. The means ± SEM of mRNA related to NC group are presented. n = 6–10. *p < 0.05, **p < 0.01, ***p<0.005, ****p<0.0005 vs NC. White bars, NC; hatched white bars, LC; hatched grey bars, NC+Ipra; black bars LC+Ipra.

To estimate glycogenolysis and glycogen synthesis in the kidney and the liver, we examined glycogen phosphorylase (*Pygl*) and glycogen synthase (*Gys1*, *Gys2*) mRNA expression. *Pygl* expression in the kidney was upregulated in the NC+Ipra group by 4.5 ± 1.5 fold compared to NC (p < 0.05) (Fig 7A), while in the liver it was upregulated in the LC group 1.8 ± 0.3 fold compared to the NC group (p < 0.01) (Fig 7C). Glycogen synthesis is conducted by muscle-type glycogen synthase *Gys1* in the kidney, but by liver-type glycogen synthase *Gys2* in the liver (Fig 3H and 3I). In the kidney, *Gys1* expression was not modulated in the NC+Ipra and LC groups, but was slightly upregulated in the combined group (Fig 7B). Similarly, in the liver, *Gys2* expression was not altered in the NC+Ipra and LC groups (Fig 7D).

### SGLT2i likely accelerate lipid metabolism in the kidney

**Fig 8:**
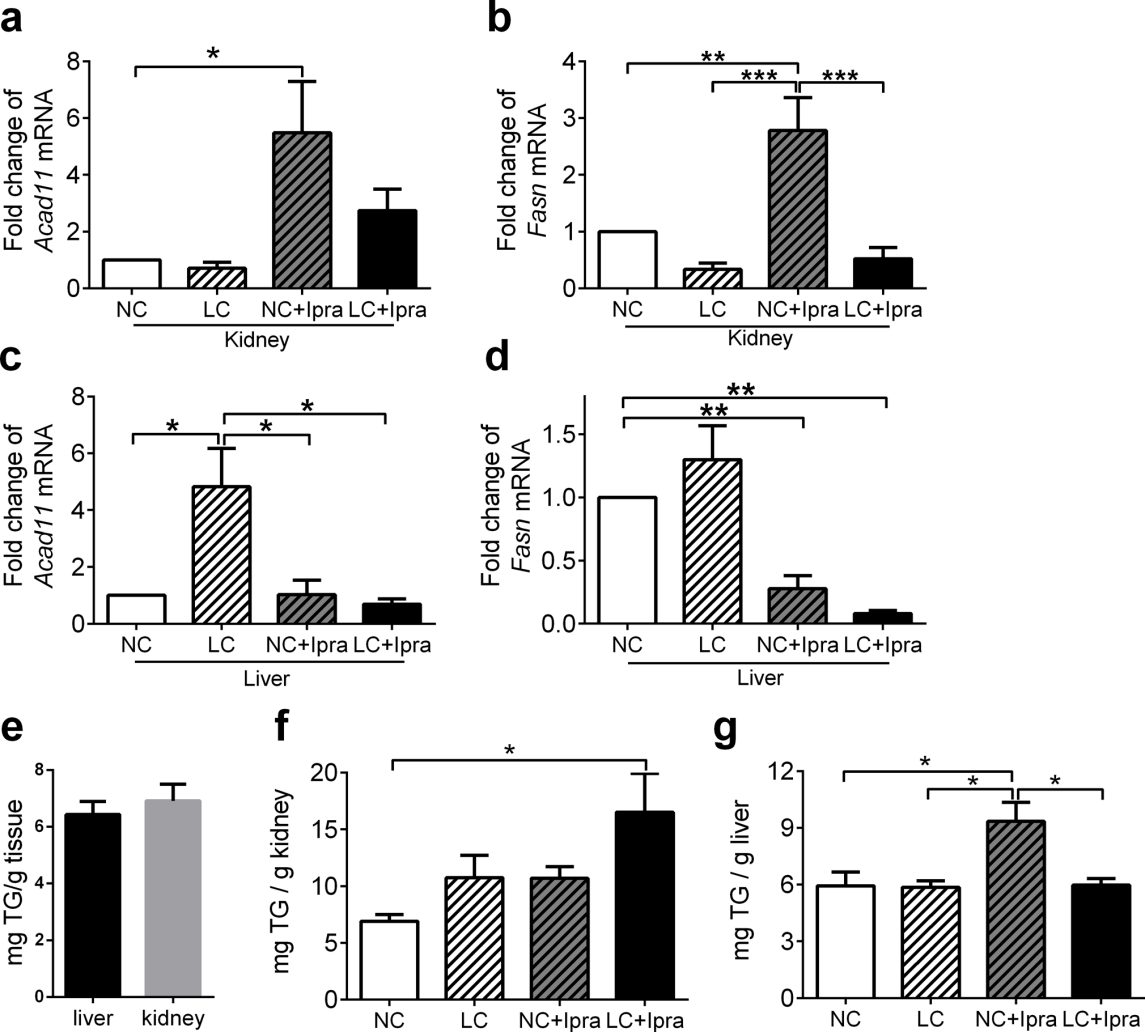
Ipragliflozin enhanced *Acad11* and *Fasn* mRNA expression in the kidney. *Acad11* and *Fasn* mRNA expression in the kidney (a, b) and liver (c, d) were determined by quantitative RT-PCR. The means ± SEM of mRNA related to NC group are presented. TG content in the kidney and the liver (e) measured in the NC group. TG content in the kidney (f) and liver (g) in non-fasted mice. n = 6–10. *p < 0.05, **p < 0.01, ***p<0.005 vs NC. White bars, NC; hatched white bars, LC; hatched grey bars, NC+Ipra; black bars LC+Ipra.

Next, we tested genes involved in β-oxidation and fatty acid synthesis. Ipragliflozin enhanced acetyl CoA dehydrogenase (*Acad11*) and fatty acid synthase (*Fasn*) mRNA expression in the kidney (Fig 8A and 8B), hence it could activate both β-oxidation and fatty acid synthesis. Interestingly, SGLT2i significantly decreased *Fasn* expression in the liver (Fig 8D), while a LCHD increased *Acad11* expression in the liver (Fig 8C).

### SGLT2i but not LCHD increased TG content in the liver

We also compared the kidney and the liver TG content among the groups. TG content in the tissues was the same in the kidney and the liver in the NC group (Fig 8E). Interestingly, in the kidney, LCHD and SGLT2i additionally increased TG content compared to the NC group (LC+Ipra: 16.5 ± 3.4 vs NC: 6.9 ± 0.6 mg TG/g kidney, p < 0.05) (Fig 8F). In the liver, TG content was significantly higher in the NC+Ipra group compared to the NC and LC groups (Fig 8G).

## Discussion

In the current study, we found that SGLT2i and LCHD could lead to different metabolic changes in non-diabetic mice. In particular, our results indicate that SGLT2i might enhance gluconeogenesis predominantly in the kidney, whereas a LCHD mainly in the liver.

SGLT2i enhanced energy intake comparable to LCHD (Fig 1C), when the animals were able to eat ad libitum, as was previously reported in animals and humans [20, 21]. The mechanism has not been well elucidated how SGLT2i increases appetite. However, it is possible that low glucose utilization (or high protein/fat utilization) will indirectly induce energy intake. Several studies indicated that some specialized neurons in the hypothalamus expresses SGLT, which is involved in glucose sensing. SGLT2i might directly affect these neurons, resulting in enhanced energy intake in mice [22–24]

We found that SGLT2i enhanced insulin sensitivity and lowered glucose excursion during OGTT (Fig 2A), although the energy utilization (subtraction of urinal disposal from oral energy intake) in the SGLT2i treated NC+Ipra group was similar to that of the NC group. It is expected that forced urinary glucose disposal might decrease postprandial insulin secretion and suppress postprandial glycemia, improving glucose tolerance [25, 26]. It was recently reported that SGLT2i tofogliflozin accelerates lipolysis in adipose tissue and improves insulin resistance chiefly in the skeletal muscle of mice fed a high-fat diet (HFD) [27]. Another SGLT2i, empagliflozin, increases glucose disposal rates, decreases hepatic glucose production and raises glucose uptake in the liver and kidney, but not in either the muscles or the adipose tissue of *db/db* mice [28]. Still, further investigations should be conducted to elucidate the precise mechanism of SGLT2i-mediated amelioration of insulin resistance.

Previous human studies have shown that a LCHD is beneficial to reduce body weight and improve insulin resistance in subjects with obesity or obese diabetes [9–11]. In contrast, we observed LCHD not only induced glucose intolerance with insulin resistance, but also enhanced adiposity in non-diabetic mice (Figs 1 and 2). Likewise, Handa et al. also reported that LCHD induces insulin resistance in normal and diabetic mice and is inversely related to the amount of dietary carbohydrate [29]. Our results could be partially explained in that the higher energy intake in LCHD led to these metabolic abnormalities. LCHD is a relative high fat diet with regards to energy composition. A high fat diet is generally believed to enhance serum TG levels and lead to fat accumulation in the liver and obesity [30–32]. Yet in our study, the LC group presented similar serum TG and NEFA levels (Table 1) and comparable hepatic TG content in the liver compared to the NC group (Fig 8G). Still, it is possible that the relative high fat composition in LCHD enhanced fat utilization, which might have played some roles in the induction of insulin resistance and obesity in our study. It can also suggest that LCHD treatment promoted the normotopic fat accumulation in the epididymal fat pad and did not induce the ectopic fat accumulation in the liver. Unlike our results, Komiya et al. reported that ipragliflozin treatment enhanced fat accumulation but ameliorated insulin resistance in epididymal adipocytes of HFD-induced obese mice [33]. Therefore, it is important to unveil the precise mechanism how the limitation of glucose utilization could enhance the fat accumulation in the different conditions.

Recent clinical studies demonstrated that treatment via SGLT2i was related to the paradoxical increase of endogenous glucose production (EGP) [26, 34] in subjects with T2DM. The treatment concomitantly increases postprandial glucagon secretion, suggesting that it might enhance gluconeogenesis and glycogenolysis in the liver. However, in our study, the mRNA of key enzymes for gluconeogenesis, *G6pc*, *Pck* and *Fbp*, was simultaneously upregulated in the kidney but not in the liver of the groups treated with ipragliflozin (Fig 4). Moreover, in the current study, long-term treatment with SGLT2i did not increase plasma glucagon levels (Table 3), though SGLT2 inhibition directly triggers glucagon secretion from pancreatic alpha cells [35].

Previous studies show that mineral corticoids or acidosis could enhance gluconeogenesis in the kidney independently from the liver [36]. Several studies have shown that gluconeogenesis is also enhanced in the diabetic kidney. The proximal tubule is considered as the only portion with appropriate enzymes for gluconeogenesis in the kidney. The proximal tubules play a key role in putting glucose influx into circulation, mostly through reabsorption via SGLTs. However, we speculate that the proximal tubules might need to accelerate gluconeogenesis to compensate for reduced glucose influx via SGLT2i. In fact, our results clearly showed that not only *Pck*, but also *G6pc* and *Fbp* mRNA expressions were simultaneously increased in the kidney of ipragliflozin-treated mice, though a report indicates that empagliflozin does not affect *Pck* mRNA expression in non-diabetic mice [37]. However, we have a limitation in our current study, since we did not directly monitor gluconeogenesis or other metabolic changes by a glucose-clamp using radioisotopes in vivo or ex vivo.

To investigate the molecular regulation of gluconeogenesis, we examined *Foxo1* and *Creb* expression and compared the expression in the kidney and the liver in mice treated with SGLT2i and/or LCHD. The expression of *Foxo1* and *Creb1* was consistently concomitant with that of *G6p*, *Pck* and *Fbp*, either in the kidney or the liver, with either SGLT2i or LCHD (Fig 5). Our results suggest that *Foxo1* and *Creb1* are key modulators of gluconeogenesis were consistently concomitant with that of *G6p*, *Pck* and *Fbp*, both in the kidney as well as the liver. It is well known that insulin and glucagon regulate expression and localization of these factors in hepatocytes [38, 39], however, further studies should be performed to know how these factors are activated in the kidney especially in the context of SGLT2i treatment.

The treatment with SLGT2i could simultaneously activate β-oxidation, and fatty acid synthesis, possibly resulting in TG accumulation in the kidney. β-oxidation was enhanced in the liver, it was only when mice were fed a LCHD (Fig 8C). It is possible that LCHD decreased utilization of glucose in the liver and that it could shift energy sources from glucose to fatty acid by enhanced β-oxidation. Meanwhile in the kidney, it is not well elucidated how SGLT2i influences β-oxidation, since fatty acids rather than glucose are the major energy source of proximal tubular cells [40, 41]

In the postabsorptive state, 55% of overall glucose release into circulation is the result of gluconeogenesis. Nevertheless, the other 45% is accounted for by glycogenolysis [42]. In our current study, SGLT2i treatment strongly reduced glycogen content in the liver, but moderately increased it in kidney (Fig 6). Glycogen is synthesized and stored abundantly in the liver and the muscles as energy storage, but also in the kidney to a small extent. A recent report suggests that the reduction of hepatic glycogen content can trigger lipolysis in the adipose tissue through the autonomic nervous system via the brain [43]. This mechanism might contribute to metabolic alterations via SGLT2i.

Here, both SGLT2i and LCHD did not affect *Gys2* expression in the liver (Fig 7D) and *Gys1* in the kidney (Fig 7B), indicating that glycogen synthesis was not greatly altered. In contrast, glycogenolytic *Pygl* expression was paradoxically increased in the kidney of the NC+Ipra group (Fig 7A) as well as in the liver of the LC group (Fig 7C). Therefore, it is still unknown why the limited glucose utilization changed hepatic or renal glycogen storage differently in SGLT2i or LCHD.

It was previously shown in diabetic rats that ‘clear cells’ store glycogen in the distal nephron segments, which are strongly affected by glucose overload in the filtrate[44, 45]. An Armani-Ebstein lesions are the abnormal synthesis and accumulation of glycogen in tubules in human diabetic patients and recognized as an early diabetic alteration. It has been recognized that abnormal glycogen accumulation is associated with persistent glucosuria. We speculate that increased reuptake in the S3 segment of the proximal tubule and luminal abnormal glucose exposure to downstream nephron segments could be the first trigger of pathological induction of glycogen accumulation in diabetes. Furthermore, this may be brought on by treatment with SGLT2is.

In conclusion, we clearly find that SGLT2i and LCHD quite distinctly influence kidney and liver metabolism. However, additional studies are necessary to investigate the mechanism in more detail.
